# Meconium pseudocyst secondary to ileum volvulus perforation without peritoneal calcification: a case report

**DOI:** 10.1186/1752-1947-4-292

**Published:** 2010-08-31

**Authors:** Esther Valladares, David Rodríguez, Antonio Vela, Sergi Cabré, Josep Maria Lailla

**Affiliations:** 1 Department of Obstetrics and Gynaecology, Hospital Sant Joan de Déu, Esplugues de Llobregat, 08950, Barcelona, Spain

## Abstract

**Introduction:**

A case of giant meconium pseudocyst secondary to ileum volvulus perforation is presented. Conventional radiographic features of meconium peritonitis with secondary meconium pseudocyst formation are well described. Our case is unusual in comparison to other cases reported in the literature and needs to be reported because the meconium pseudocyst presented without the typical ultrasound features (calcifications, polyhydramnios and ascites) and was initially identified as an abdominal mass.

**Case presentation:**

We describe the case of a 29-year-old Caucasian woman in her third trimester of pregnancy, in which an abdominal mass was detected in the fetus. The newborn was diagnosed in the early neonatal period with meconium pseudocyst secondary to ileum volvulus perforation.

**Conclusions:**

The prenatal appearance of a meconium pseudocyst can be complemented by other signs of bowel obstruction (if present) such as polyhydramnios and fetal bowel dilatation. This is an original case report of interest to all clinicians in the perinatology and fetal ultrasound field. We consider that the utility of this case is the recognition that a meconium pseudocyst might appear without the typical ultrasound features and should be considered as a differential diagnosis when an echogenic intra-abdominal cyst is seen.

## Introduction

Intra-uterine intestinal perforation causes a sterile inflammatory reaction of the peritoneum known as meconium peritonitis.

The ultrasound diagnosis of meconium peritonitis should be considered in the presence of a fetal intra-abdominal hyper-echoic mass, particularly if associated with ascites and polyhydramnios. Meconium cysts usually contain characteristic punctate echogenic calcifications as well.

With technical advances in imaging and increasing use of high-resolution ultrasonic equipment, a significant number can now be diagnosed prenatally. Magnetic resonance imaging may also be a valuable diagnostic tool.

Meconium pseudocyst secondary to ileum volvulus perforation is an uncommon cause of fetal abdominal mass. We report an unusual case of meconium pseudocyst presenting without the typical features identified on ultrasound examination.

## Case presentation

A 29-year-old Caucasian woman with a 32.3 week, twin bicorial biamniotic pregnancy was admitted to the Emergency Service with threat of preterm labor. Tocolysis with atosiban and fetal lung maturation pattern were provided.

Social and medical history were remarkable for gestational diabetes and a previous evaluation for primary sterility through laparoscopy and hysteroscopy, but were otherwise non-contributory.

**Figure 1:**
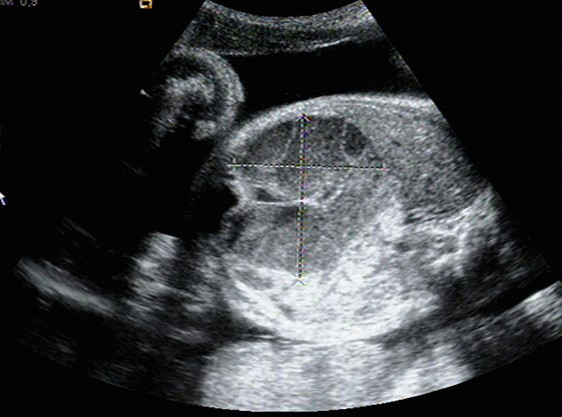
**Ultrasound image: 32.3 weeks of gestation**. Transverse scan image thorough the fetal abdomen identifying a mass occupying the entire left hemiabdomen (meconium pseudocyst), with mixed echogenicity. No calcifications were observed.

**Figure 2:**
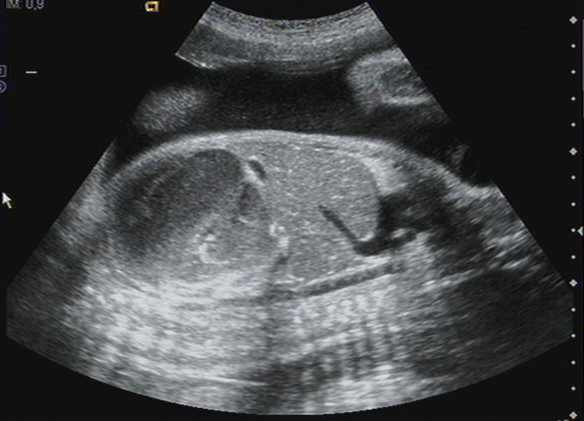
**Ultrasound image: 32.3 weeks of gestation**. Longitudinal scan image thorough the fetal abdomen identifying a mass occupying the entire left hemiabdomen (meconium pseudocyst), with mixed echogenicity. No calcifications were observed.

The first day of hospitalization (32.3 weeks), third trimester fetal ultrasound was performed. An abdominal mass occupying the entire left hemiabdomen with mixed echogenicity was identified in the first fetus (cephalic presentation) (Figures 1 and 2). No calcifications were observed. The fetus's stomach and amniotic fluid volume were normal. Neuroblastoma or meconium pseudocyst were suspected. The first fetus had abnormal umbilical artery and normal middle cerebral artery Doppler studies. The second fetus (transverse situation) had no apparent pathology and normal Doppler studies. Previous ultrasound examinations of both fetuses before 32 weeks were normal.

**Figure 3:**
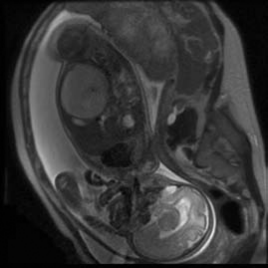
**Magnetic resonance imaging: 32.4 weeks of gestation**. Longitudinal magnetic resonance image of the fetus demonstrating the meconium pseudocyst; a 72 × 58 mm, heterogeneous, mesenteric mass without necrosis causing significant distortion of the small intestine to the left. No calcification or ascites were observed.

**Figure 4:**
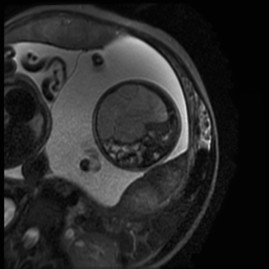
**Magnetic resonance imaging: 32.4 weeks of gestation**. Transverse magnetic resonance image of the fetus demonstrating the meconium pseudocyst; a 72 × 58 mm, heterogeneous, mesenteric mass without necrosis causing significant distortion of the small intestine to the left. No calcification or ascites were observed.

Fetal magnetic resonance imaging (performed at 32.4 weeks) identified a 72 × 58 mm, heterogeneous, mesenteric mass without necrosis causing significant distortion of the small intestine to the left. There were no pathologic findings in the rest of abdominal structures (Figures 3 and 4). There were no calcifications, ascites, polyhydramnios or bowel loop dilatation.

At 32.6 weeks of gestation, uterine contractions and cervical ripening began. Urgent cesarean section was performed due to preterm labor associated with fetal malposition (transverse situation). Birth weights were 1980 g (fetus 1) and 2060 g (fetus 2).

Laparotomy and bowel resection were performed within the first day following delivery. During surgery a 10 cm, volvulated, necrotic portion of small intestine was identified, at 10 cm from ileocecal valve. Small bowel volvulus resection, termino-terminal anastomosis, and appendectomy were performed.

Pathological anatomy reports revealed distal ileum vascular congestion, intestinal wall bleeding and areas of acute inflammation.

The final diagnosis was a perforated ileum volvulus and secondary meconium pseudocyst.

Bowel obstruction was suspected at three days following the initial surgical intervention. A second laparotomy identified a segment of obstructed bowel. This was resected and a termino-terminal re-anastomosis was performed. Sweat chloride test for cystic fibrosis was negative.

Due to the newborn's torpid post-operative course and lack of gastrointestinal tolerance, an exploratory laparotomy was performed 51 days after birth. Intra-operatively, a stenosis of the re-anastomosis was observed. Resection of a 5 cm section of bowel including ileocecal valve, as well as ileostomy and colostomy were performed.

The newborn remained hospitalized receiving total parenteral nutrition and with secretory diarrhea due to short bowel syndrome, and died during the seventh month of life.

## Discussion

The differential diagnosis of a sonographically visualized intra-abdominal cyst in a fetus is extensive, and includes intestinal duplications cyst, mesenteric cysts, choledochal cyst, meconium pseudocyst, congenital cyst of the pancreas, renal cyst, obstructive uropathy, urachal cyst, ovarian cyst, ureterocele and tumorous lesions such as cystic sacroccocygeal teraromas.

Fetal tumors comprise 0.5% to 2% of all childhood neoplasms. Extra-cranial teratomas, neuroblastomas, soft-tissue and intra-cranial tumors are the most common (85% of all tumors). The remaining 15% are made up of renal tumors, liver tumors, retinoblastoma and other less common processes that can mimic a tumor, such as meconium peritonitis (cystic type) [1].

Meconium peritonitis is a sterile chemical peritonitis caused by meconium extruding into the peritoneal cavity through a small bowel perforation *in utero*. The estimated prevalence is about 0.29 per 10,000 live births and the mortality ranges from 11% to 50%. It usually appears in the neonatal period with abdominal distension, vomiting, acidosis and intra-abdominal calcifications.

Perforation occurs most commonly in the ileum proximal to an obstruction, but this cannot always be demonstrated. The obstruction can be caused by atresia, stenosis, volvulus, internal bowel hernia, Meckel's diverticulum, meconium ileus, or peritoneal bands. Intestinal stenosis or atresia and meconium ileus account for 65% of the cases. Adhesions between loops of intestine and omentum act to contain the meconium collection extruded into the peritoneal cavity, creating a cystic mass that can be visualized on ultrasound. The reaction may alternatively result in the formation of a solid non-cystic mass with calcium deposits sealing off the perforation [2]. When the formation of this apparently solid abdominal mass occurs, an accurate diagnosis between an abdominal tumor and meconium collection may be challenging.

In a review of 12 cases of meconium peritonitis, intra-peritoneal calcifications were present in 60% of the patients with cystic fibrosis and 100% of patients without cystic fibrosis [3]. The authors postulate that pancreatic enzymes, which are in a low concentration in 80% of patients with cystic fibrosis, may be necessary for the calcifications to occur. Our case showed no evidence of cystic fibrosis. It is possible that the ultrasound was performed soon after the creation of the pseudocyst and before the calcification could be visible sonographically. Calcifications can develop within days, but may need several weeks to be visible sonographically [3].

Cystic fibrosis is the most common fatal autosomal recessive disease among Caucasian population, with a frequency of one in 2000 to 3000 live births. The sweat chloride test remains the primary test for the diagnosis of this disease; the DNA testing is used for confirmation of patients with intermediate sweat chloride results. The sweat testing is performed by the collection of sweat with pilocarpine iontophoresis, and chemical determination of the chloride concentration [4]. Meconium ileus is the presenting problem in 10 to 20 percent of newborns with cystic fibrosis, and is virtually pathognomonic of the disease. Volvulus in fetal life is suggestive of cystic fibrosis; episodes of small bowel obstruction may also occur in older children and adults.

Depending on when the bowel perforation occurs during development and the severity of the inflammatory reaction induced by the meconium extruded into the peritoneal cavity, three different types of meconium peritonitis can be described according to the ultrasound findings [5]. The fibroadhesive type is the most frequent and is characterized by an intense fibroblastic reaction causing the formation of fibrotic membranes which are adherent to the intestinal wall and cover the perforation. Ultrasound reveals the presence of diffuse punctiforme hyper-echogenic lesions around the peritoneal cavity. Intra-abdominal calcifications are not usually observed. Ascitis, hydramnios or bowel loop dilatation are also characteristic. The perforation may not be visualized as it often seals spontaneously. The cystic type, as found in the present case, is formed by a meconium collection surrounded by fibrotic membranes (pseudocyst). Through ultrasound imaging the pseudocyst appears as a large meconium-filled cyst lined by a thick membrane containing multiple calcium deposits and plaques. The cystic type is usually formed secondary to a prenatal volvulus with perforation [6]. The last category is the generalized type, and is the consequence of a peri-natal perforation with meconium spread throughout the abdominal cavity.

One study [7] has described the relationship between ultrasound findings and the post-natal course of meconium peritonitis. A total of 69 cases were divided into four grades according to their ultrasound features. Grade 0, isolated intra-abdominal calcifications; grade 1, intra-abdominal calcifications and ascites or pseudocyst or bowel dilatation; grade 2, two associated findings; grade 3, all sonographic features. The authors found an increasing need for neonatal surgery with higher grades of the sonographic classification [7]. Another study also found a correlation between ultrasound features and clinical implications [8]. Persistent ascites, pseudocyst or dilated bowel loop were reported to be the most sensitive predictors of post-natal surgery (92%, *P *< 0.022) [9].

Meconium pseudocysts are often accompanied by polyhydramnios [10]. It is often the consequence of associated bowel atresia or extrinsic mechanical obstruction of the bowel due to mass effect. A large fetal intra-abdominal mass may additionally cause fetal lung immaturity; however, percutaneous drainage of these cysts may cause leakage of the meconium into the amniotic fluid.

The MR appearance of meconium pseudocysts have been described in the literature [11,12].

With one exception, all cases of meconium pseudocyst were associated with bowel dilatation or free intra-abdominal fluid [13]. In another case [14], the meconium pseudocyst was associated with dilated bowel and ascites, but had no calcifications in a newborn with a normal sweat test. A separate study describes 11 cases of meconium peritonitis [15]. In one case from this study which was similar to ours, the only ultrasound finding was a meconium pseudocyst. In nine other cases, the meconium pseudocyst was associated with polyhydramnios, ascites or dilated bowel loops. In the remaining case, fetal ascites was the only ultrasound finding.

Treatment for meconium pseudocyst usually consists of surgical resection, although definitive procedures in the early neonatal period are usually difficult. Consequently, many patients require more than one surgical intervention. Some authors recommend immediate cyst drainage and decompression through paracentesis following birth with delayed definitive resection [16]. The prognosis was poor in the past, but has improved due to the development of newer surgical techniques. Eckoldt [15] demonstrated a successful management with patient survival in nine out of 11 cases. In cases with underlying atresia, temporary diversion enterostomy with planned secondary reconstruction at two to three weeks showed good results. For large meconium pseudocysts, a two-stage approach with cyst decortication and temporary enterostomy, followed by elective reversal is the gold standard.

## Conclusions

Meconium peritonitis is an uncommon fetal and neonatal condition and it should be considered in the differential diagnosis when an echogenic intra-abdominal mass is observed. The prenatal appearance can be accompanied by signs of bowel obstruction, such as polyhydramnios and bowel dilatation. Generalized hydrops increases the severity of this disease.

Surgery should be performed as soon as possible after delivery and initial resuscitation although immediate decompression paracentesis may result in a rapid improvement in the overall state of the newborn while preparation for surgery is underway. A two stage-approach with temporary enterostomy and delayed reversal is the best choice.

Our case is unusual in comparison to other sonographically described prenatal cases due to the large size of the pseudocyst, the absence of ascites, bowel dilatation, or polyhydramnios, as well as a lack of abdominal calcifications in a newborn without cystic fibrosis.

The clinical utility of this case is the recognition that meconium pseudocyst may present without typical ultrasound features, and should be considered in the differential diagnosis of an abdominal mass. This will facilitate delivery of appropriate treatment as soon as possible after birth.

## Competing interests

The authors declare that they have no competing interests.

## Consent

Written informed consent was obtained from the patient for both her case and the case of her child for publication of this case report and any accompanying images. A copy of the written consent is available for review by the Editor-in-Chief of this journal.

## Authors' contributions

EV collected the clinical case, wrote the manuscript, and conducted the literature search. DR collected previous similar clinical cases from the literature, drafted the manuscript, and attended the discussion. AV visited the patient, made the ultrasound diagnosis, and gave advice on the literature search. SC developed the article concept, provided ultrasound images and contributed to writing the introduction. JML provided general supervision and analyzed and interpreted the patient data. All authors have read and approved the final manuscript
